# Endocytosis-Independent Function of Clathrin Heavy Chain in the Control of Basal NF-κB Activation

**DOI:** 10.1371/journal.pone.0017158

**Published:** 2011-02-25

**Authors:** Man Lyang Kim, Isabel Sorg, Cécile Arrieumerlou

**Affiliations:** Biozentrum, University of Basel, Basel, Switzerland; Thomas Jefferson University, United States of America

## Abstract

**Background:**

Nuclear factor-κB (NF-κB) is a transcription factor that regulates the transcription of genes involved in a variety of biological processes, including innate and adaptive immunity, stress responses and cell proliferation. Constitutive or excessive NF-κB activity has been associated with inflammatory disorders and higher risk of cancer. In contrast to the mechanisms controlling inducible activation, the regulation of basal NF-κB activation is not well understood. Here we test whether clathrin heavy chain (CHC) contributes to the regulation of basal NF-κB activity in epithelial cells.

**Methodology:**

Using RNA interference to reduce endogenous CHC expression, we found that CHC is required to prevent constitutive activation of NF-κB and gene expression. Immunofluorescence staining showed constitutive nuclear localization of the NF-κB subunit p65 in absence of stimulation after CHC knockdown. Elevated basal p65 nuclear localization is caused by constitutive phosphorylation and degradation of inhibitor of NF-κB alpha (IκBα) through an IκB kinase α (IKKα)-dependent mechanism. The role of CHC in NF-κB signaling is functionally relevant as constitutive expression of the proinflammatory chemokine interleukin-8 (IL-8), whose expression is regulated by NF-κB, was found after CHC knockdown. Disruption of clathrin-mediated endocytosis by chemical inhibition or depletion of the μ2-subunit of the endocytosis adaptor protein AP-2, and knockdown of clathrin light chain a (CHLa), failed to induce constitutive NF-κB activation and IL-8 expression, showing that CHC acts on NF-κB independently of endocytosis and CLCa.

**Conclusions:**

We conclude that CHC functions as a built-in molecular brake that ensures a tight control of basal NF-κB activation and gene expression in unstimulated cells. Furthermore, our data suggest a potential link between a defect in CHC expression and chronic inflammation disorder and cancer.

## Introduction

Nuclear factor-kappa B (NF-κB) transcription factors control the expression of genes involved in a large spectrum of biological processes, including inflammation, adaptive immunity, stress responses, angiogenesis, cell proliferation and invasion [1], [2]. Aberrant regulation of NF-κB activity has been associated with immune disorders and numerous cancers [3]. Although NF-κB has been the subject of intensive investigation, the molecular mechanisms underlying its regulation are not fully understood. There are five NF-κB isoforms in mammalian cells: p65/RelA, RelB, c-Rel, p50 (NF-κB1) and p52 (NF-κB2). All these proteins share a Rel homology domain responsible for homo- and heterodimerization as well as for sequence-specific DNA binding. Among the various hetero-and homodimers formed by NF-κB proteins, the p50/p65 heterodimer is predominant in many cell types [4]. Dimers of NF-κB proteins bind κB sites in promoters or enhancers of target genes and regulate transcription via the recruitment of transcriptional co-activators and co-repressors. A number of posttranslational modifications of the NF-κB proteins, including phosphorylations and acetylations, further modulate DNA binding and, therefore, transcriptional activity [5]. In absence of stimulation, most of the NF-κB dimers are retained in the cytoplasm by the inhibitor of NF-κB (IκB) family members whose prototype is the protein IκBα [4], [6], [7]. IκBα contains several ankyrin repeats that mediate the binding to NF-κB dimers and mask the nuclear localization signal (NLS) of p65. Following cell stimulation by proinflammatory cytokines, such as tumor necrosis factor α (TNFα) and interleukin-1, IκBα is rapidly phosphorylated on serine 32 and serine 36 residues by the IκB kinase (IKK) complex composed of three subunits: two catalytic subunits, IKKα and IKKβ, and the regulatory scaffold component NF-κB essential modulator (NEMO). IκBα phosphorylation is then followed by rapid polyubiquitination and degradation via the 26S proteasome. Released NF-κB dimers translocate into the nucleus where they drive gene expression [8], [9]. As the gene encoding IκBα is rapidly upregulated following NF-κB activation, IκBα is promptly resynthesized [10]. Newly synthesized IκBα proteins bind to nuclear NF-κB dimers and dissociate them from DNA. This mechanism terminates the transcriptional activity of NF-κB and resets gene expression to basal level.

Although constitutive NF-κB activation has been associated with inflammatory disorders and numerous cancers [3], [11], the mechanisms leading to elevated basal NF-κB activation remain unclear. Proposed mechanisms include activation of kinases, overexpression of cytokines, dysregulation of cell surface receptors and activation of oncoproteins. We recently performed an RNA interference (RNAi) screen targeting host signaling proteins that could potentially be involved in the inflammatory response following infection by *Shigella flexneri* [12]. From that screen, we identified clathrin heavy chain (CHC) as one of the proteins that, when knocked down, strongly enhanced activation of NF-κB. In this study, we examine the role of CHC in the control of basal NF-κB activation.

CHC is mainly known as a structural component of clathrin and for its role in clathrin-mediated endocytosis (CME) [13], [14]. The association of three CHCs and up to three clathrin light chains (CLCs) forms a clathrin triskelion structure that self-polymerizes to form a curved lattice around invaginated pits. Through this mechanism, CHC is involved in the uptake of nutrients, the internalization of pathogens, the downregulation of certain ligand-induced receptors and in protein sorting at the trans-Golgi network (TGN) during protein secretion [13], [15], [16]. However, similar to other endocytic proteins [17], CHC appears to perform multiple functions in cells. It has been reported that CHC is involved in chromosome segregation during mitosis [18]. In addition, a fraction of CHC proteins that localize to the nucleus bind to the p53-responsive promoter and favor p53-mediated transcription [19].

Here we have used RNAi to effectively knock down CHC in epithelial cells. Surprisingly, we found that the depletion of CHC induces constitutive nuclear localization of the NF-κB subunit p65 in absence of stimulation. Elevated basal p65 nuclear localization was associated with constitutive phosphorylation and degradation of IκBα via an IKKα dependent mechanism and constitutive expression of the proinflammatory chemokine interleukin-8 (IL-8), whose expression is regulated by NF-κB. Interestingly, CHC acted on NF-κB independently from its roles in CME and from CLCs. Taken together, our data reveal a new function of CHC in the control of basal NF-κB activity and gene expression in epithelial cells.

## Materials and Methods

### Antibodies and reagents

Antibodies against NF-κB p65, IκBα, CLCa and IKKα were obtained from Santa Cruz Biotechnology (Santa Cruz, USA) while the CHC antibody was from BD Transduction Laboratories (San Jose, USA). The actin antibody was from Chemicon (Billerica, USA) and the phospho-IκBα antibody was from Cell signaling technology (Beverly, USA). The anti-mouse IgG-Cy5 was obtained from Zymed (San Francisco, USA) and the anti-rabbit IgG-HRP and anti-mouse IgG-HRP from GE Healthcare (Pittsburgh, USA). Hoechst 33342 and FITC-phalloidin were from Invitrogen (Carlsbad, USA).

### Cell culture and siRNA transfection

HeLa Kyoto [20] and MCF-7 cells (ATCC, Manassas, USA) were maintained in Dulbecco's modified Eagle's medium (high glucose) supplemented with 10% fetal bovine serum, 100 units/ml penicillin, and 100 µg/ml streptomycin at 37°C in 10% CO_2_. HeLa and MCF-7 cells were transfected with different siRNAs at 10 nM using Lipofectamine 2000 (Invitrogen, Carlsbad, USA). ON-TARGETplus SMARTpool siRNAs for clathrin heavy chain (CHC/CLTC, #L-004001-00-005), clathrin light chain a (CLCa/CLTA, #L-004002-00-005), AP2M1 (#L-008170-00-005), IKKα (#L-003473-00-005) and ON-TARGETplus siCONTROL were obtained from Dharmacon (Dallas, USA).

### Immunofluorescence and microscopy

Cells were fixed with 4% PFA for 6 min and permeabilized in 0.5% Triton X-100 for 10 min. They were, then, incubated with a mouse monoclonal p65 antibody (1 µg/ml) overnight at 4°C and stained with a Cy5-conjugated secondary antibody and Hoechst (10 µg/ml) for 40 min at room temperature. Images were acquired at 12 random sites of each well using the automated ImageXpress microscope (Molecular devices, Sunnyvale, USA). The nuclear localization of p65 was automatically quantified by using the Enhanced-Translocation module of MetaXpress (Molecular devices, Sunnyvale, USA). Briefly, the Hoechst staining was used as a mask to automatically identify nuclei in the p65 staining image. The cytoplasmic area of each cell was defined by a ring around the nucleus. For each cell, the ratio of p65 intensity in the nucleus and in the cytoplasmic ring defined as the Nuc/Cyt p65 NF-κB ratio was calculated and averaged over several thousands of cells per well.

### Transferrin uptake assay and inhibition of endocytosis

Transferrin uptake was measured as described by Galvez et al [21]. Briefly, HeLa cells were treated with Alexa 594-conjugated transferrin (Invitrogen) for 10 min followed by a quick acid wash to cleave off the receptor-bound transferrin from the plasma membrane. Cells were then fixed with 4% PFA and stained with Hoechst. Transferrin uptake was automatically quantified by using the Multi-wave Length Cell Scoring module of MetaXpress (Molecular devices, Sunnyvale, USA). To inhibit endocytosis, cells were pretreated with 80 µM dynasore (Sigma) or 5 µM phenylarsine oxide (PAO) (Sigma) in complete growth medium for the indicated time periods. The inhibitor concentration was kept constant during the assays. Inhibition of endocytosis was verified by measuring the uptake of transferrin as described above. For 48 hour of drug treatment, the solution of complete growth medium containing dynasore was replaced after 24 hours with a fresh solution. To verify the activity of dynasore after 24 hour of incubation, the uptake of transferrin was measured in HeLa cells treated with a solution of dynasore incubated in growth medium for 24 hours.

### Enzyme-linked Immunosorbent Assay (ELISA)

IL-8 secretion was measured by ELISA in the supernatant of siRNA-transfected HeLa and MCF-7 cells, 72 hours post transfection. Cell-free supernatants from triplicate wells were analyzed for their IL-8 content using a commercial ELISA kit (BD Pharmingen, San Jose, USA). In parallel, cells from the plate were stained with Hoechst to quantify cell numbers. IL-8 measurements were normalized to the number of cells for each condition.

### Western Blot Analysis

HeLa or MCF-7 cells were transfected with siRNAs in a 6-well plate. 72 hours post transfection, cells were lysed in Phosphosafe Extraction Buffer (Novagen, Darmstadt, Germany) supplemented with 1× protease inhibitor cocktail (Calbiochem, Darmstadt, Germany). Protein concentration was measured using the bicinchoninic acid (BCA) kit (Pierce, Rockford, USA). Equal amounts of proteins were resolved by SDS-PAGE and transferred to Hybond C-Extra membrane (Amersham Bioscience, Pittsburgh, USA) for immunoblotting with indicated antibodies. Primary antibodies were detected using horseradish peroxidase-conjugated anti-rabbit or anti-mouse IgG antibodies, and visualized with the ECL system (Pierce). Quantification of the blots was performed using the densitometry feature of Photoshop.

### Quantitative real-time PCR

Total RNA was isolated from control or CHC siRNA transfected cells in a 6-well plate using the total RNA purification system (Invitrogen, Carlsbad, USA). cDNAs were generated by using the Superscript III 1st Strand Synthesis Kit (Invitrogen, Carlsbad, USA). Real-time PCR was performed on an ABI Prism 7700 system (Applied Biosystems, Foster city, USA) using the SYBR green PCR Master Mix (Applied Biosystems, Foster city, USA) to measure relative IκBα mRNA level in CHC-depleted and control cells. GAPDH was used as an internal control to normalize mRNA expression. Each sample was analyzed in triplicate. The primer sequences used are as follows. IκBα-forward: 5′-GACCTGGTGTCACTCCTGTTG; IκBα-reverse: 5′-CTCTCCTCATCCTCACTCTCTGG; GAPDH-forward: 5′-GAAGGTGAAGGTCG GAGTC; GAPDH-reverse: 5′-GAAGATGGTGATGGGATTTC.

### Statistical analysis

Results are expressed as the mean ± SD as specified in figure legends. p values were calculated with a two-tailed two-sample equal variance t-test. p values of less than 0.05 were considered statistically significant.

## Results

### CHC prevents constitutive NF-κB p65 nuclear localization in unstimulated epithelial cells

**Figure 1:**
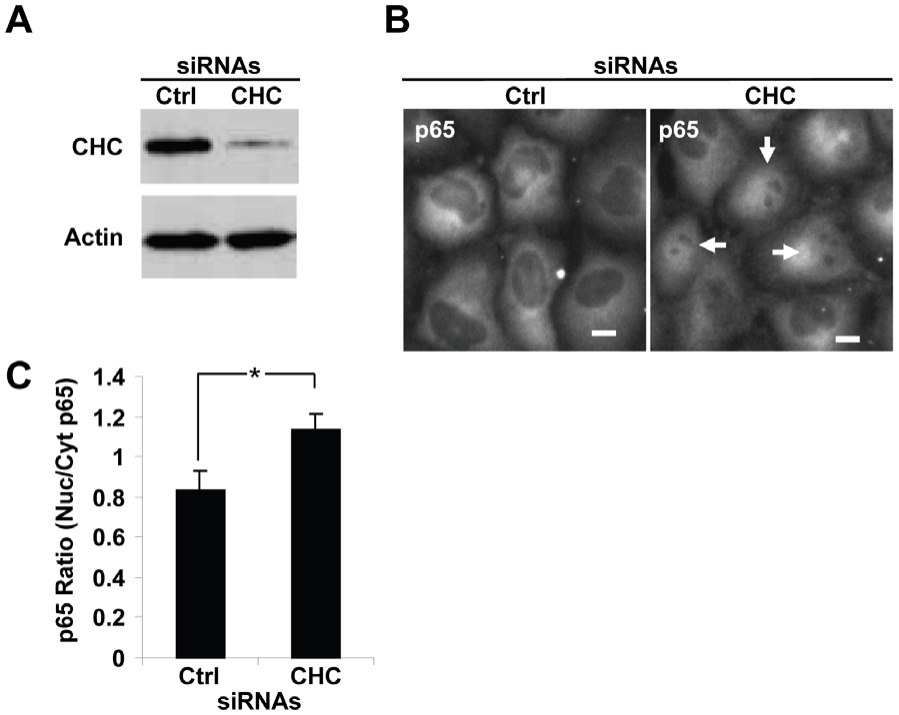
CHC prevents constitutive NF-κB p65 nuclear localization in unstimulated epithelial cells. (**A**) Effective knockdown of CHC after siRNA transfection. Lysates from HeLa cells transfected with control (Ctrl) or CHC siRNAs for 72 hours were analyzed by western immunoblotting using indicated antibodies. Actin is shown as a loading control. (**B**) Constitutive nuclear localization of p65 after CHC knockdown. HeLa cells were transfected with either control or CHC siRNA and p65 localization was visualized by immunofluorescence microscopy. White arrows indicate cells showing a clear nuclear localization of p65. Scale bars, 10 µm. (**C**) Quantification of the nuclear/cytosolic p65 intensity ratio in control and CHC siRNA transfected HeLa cells (results are expressed as the mean ± SD of 12 images; *p = 3.14E-07, graph representative of 3 independent experiments).

To investigate the implication of CHC in the regulation of basal NF-κB activation, we tested whether CHC interfered with the localization of the NF-κB subunit p65 in absence of stimulation. For this purpose, HeLa cells were depleted of CHC by transfection with a pool of four siRNAs targeting CHC. A pool of four none-targeting siRNAs was used as control in parallel. The efficiency of knockdown after 72 hours was controlled by measuring the expression of CHC by western immunoblotting (Figure 1A). The localization of p65 was visualized by immunofluorescence microscopy with an anti-p65 antibody. As expected, p65 was mostly present in the cytoplasm of control cells (Figure 1B, left panel). Surprisingly, both cytoplasmic and nuclear localization of p65 was observed after CHC knockdown (Figure 1B, right panel). This observation was confirmed by quantification with automated image processing of the nuclear/cytoplasmic p65 intensity ratio (Figure 1C). These results showed that CHC expression is required to prevent constitutive nuclear localization of p65 in unstimulated HeLa cells.

### CHC prevents constitutive IKK-mediated phosphorylation and degradation of IκBα in unstimulated epithelial cells

**Figure 2:**
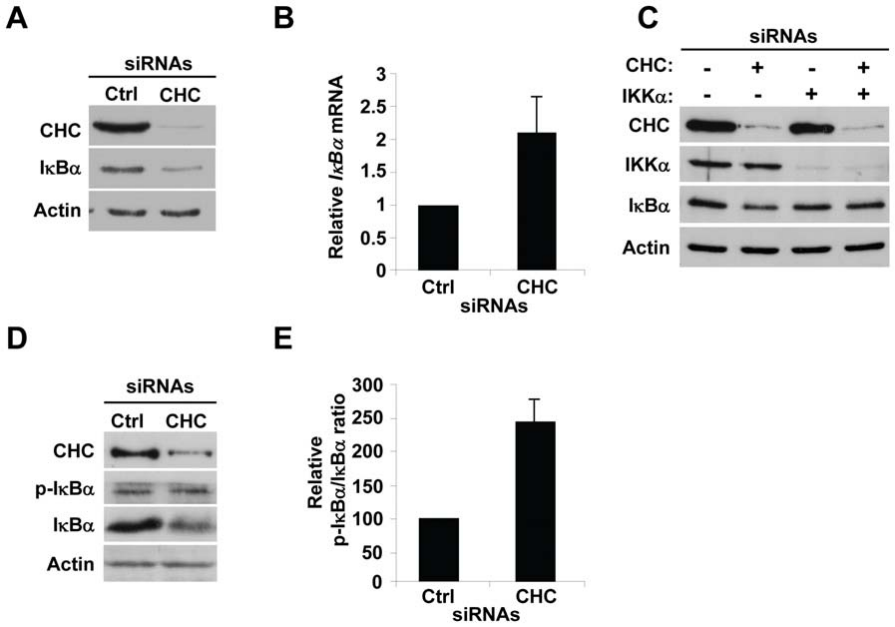
CHC prevents constitutive degradation and phosphorylation of IκBα by an IKKα-dependent mechanism. (**A**) Reduced level of IκBα after CHC knockdown in HeLa cells. Cell lysates from control or CHC siRNA transfected cells were analyzed by western immunoblotting using indicated antibodies. Actin is shown as a loading control (representative of 3 independent experiments). (**B**) Quantification of the level of *IκBα* mRNA by quantitative RT-PCR in control or CHC-depleted HeLa cells. *GAPDH* mRNA was used as an internal control for normalization (results are expressed as the mean ± SD of 3 independent experiments). (**C**) IKKα-depletion abolishes the constitutive degradation of IκBα induced by CHC knockdown. Lysates from cells transfected with different combinations of IKKα and CHC siRNAs were analyzed by immunoblotting using indicated antibodies. Total siRNA concentration was kept constant by adding appropriate amounts of control siRNAs. Actin is shown as a loading control (data representative of 2 independent experiments). (**D**) CHC prevents enhanced basal phosphorylation of IκBα at position serine 32. Lysates from control or CHC-depleted HeLa cells were analyzed by immunoblotting using the indicated antibodies. Actin is shown as a loading control. (**E**) Densitometric quantification of the p-IκBα/IκBα ratio (results are expressed as the mean ± SD of 3 independent experiments).

The localization of p65 results from a complex equilibrium between cytoplasm to nucleus translocation and nuclear export [10]. We analyzed, most specifically, the implication of CHC in the mechanisms that control the nuclear translocation of p65. In the canonical NF-κB activation pathway triggered by most stimuli, this process is tightly controlled by IKK complex-dependent phosphorylation and proteolytic degradation of IκB proteins. As we observed more p65 in the nuclei of CHC-depleted cells, we hypothesized that basal IκB degradation was elevated in these cells. To directly test this assumption, the level of IκBα in CHC and control siRNA transfected cells was analyzed by western immunoblotting. As shown in Figure 2A, a strong reduction in the level of IκBα was found after CHC knockdown. To exclude the hypothesis that this diminution resulted from reduced *IκBα* gene transcription, the level of IκBα mRNA was analyzed by quantitative real-time PCR. A two-fold increase in IκBα mRNA was measured after knockdown compared to control (Figure 2B), indicating that the reduction of IκBα level was not caused by an inhibition of transcription but, most likely, by constitutive degradation of IκBα proteins. Because the catalytic subunit IKKα largely contributes to IκBα phosphorylation and degradation in HeLa cells [22], we examined the effect of IKKα knockdown on constitutive IκBα degradation. For this purpose, HeLa cells were transfected with combinations of CHC and IKKα siRNAs for single or co-depletion experiments as described in Figure 2C. When IKKα was depleted, the knockdown of CHC had no effect on the level of IκBα (Figure 2C), showing that CHC controls basal IκBα degradation by a mechanism dependent on IKKα IκBα proteins are subject to phosphorylation by the IKK complex prior to degradation. Therefore we tested if constitutive IκBα degradation resulted from an increase of IκBα phosphorylation (p-IκBα). The phosphorylation at position serine 32 was analyzed by western immunoblotting using a phospho-specific antibody. Interestingly, a two-fold increase in the level of p-IκBα was observed in CHC-depleted cells (Figures 2D and 2E) after normalization to the level of IκBα, suggesting that constitutive degradation of IκBα was caused by constitutive IκBα phosphorylation. Taken together, these results showed that CHC prevents constitutive NF-κB activation in unstimulated HeLa cells by blocking the spontaneous phosphorylation and degradation of IκBα by the IKK complex.

### CHC prevents constitutive IL-8 secretion in unstimulated epithelial cells

**Figure 3:**
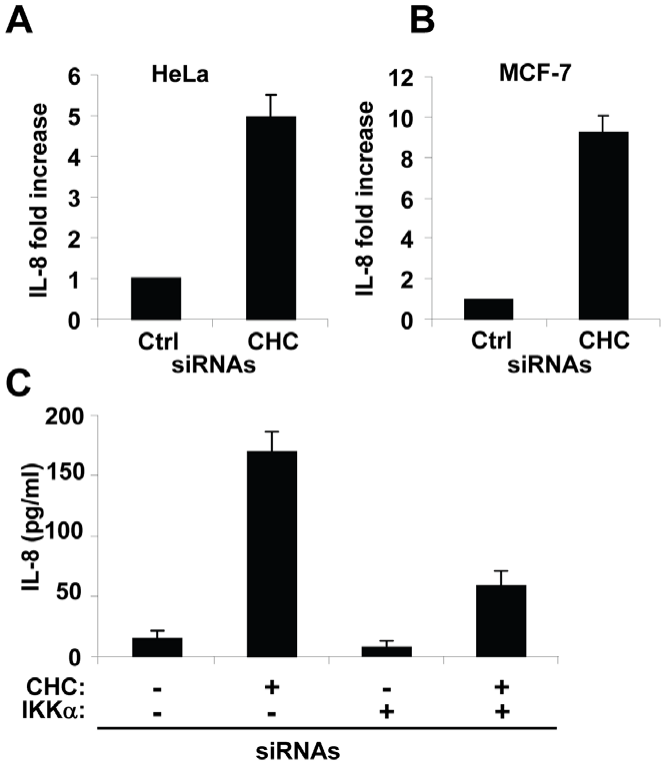
CHC prevents constitutive IL-8 expression in unstimulated epithelial cells. (**A**) Constitutive IL-8 expression after knockdown of CHC in HeLa cells. Cells were transfected with control or CHC siRNAs. After 72 hours, supernatants were collected and analyzed for their content in IL-8 by ELISA (results are expressed as the mean ± SD of 3 independent experiments). (**B**) Constitutive IL-8 expression after knockdown of CHC in MCF-7 cells. MCF-7 cells were treated as described in (A) (results are expressed as the mean ± SD of 3 independent experiments). (**C**) IKKα-depletion abolishes the constitutive secretion of IL-8 induced by CHC knockdown. HeLa cells were transfected with different combinations of IKKα and CHC siRNAs for 72 hours. Total siRNA concentration was kept constant by adding appropriate amounts of control siRNAs. Supernatants were collected to measure the concentration of IL-8 by ELISA (results are expressed as the mean ± SD of 3 independent experiments).

Previous results indicated that CHC was required to prevent constitutive p65 nuclear translocation. Because this process directly contributes to the regulation of gene expression, we tested whether the presence of CHC was also necessary to prevent constitutive expression of genes regulated by NF-κB. In particular, we investigated the expression of the proinflammatory chemokine IL-8. IL-8 secretion was measured by ELISA in the supernatant of CHC and control siRNA transfected HeLa cells. In line with the results obtained on NF-κB activation, knocking down CHC strongly enhanced basal IL-8 secretion (Figure 3A), showing that, indeed, the expression of CHC was critical to prevent constitutive IL-8 expression in HeLa cells. The same result was obtained in the breast cancer cell line MCF-7 (Figure 3B). Furthermore, consistent with the results obtained on IκBα degradation, constitutive expression of IL-8 was massively reduced when IKKα was knocked down (Figure 3C). Taken together, these results showed that CHC prevents constitutive expression of IL-8, and that this new function of CHC in NF-κB signaling depends on IKKα and corresponds to a general mechanism taking place in different cells lines.

### CHC controls basal NF-κB activation independently of endocytosis and clathrin light chains

**Figure 4:**
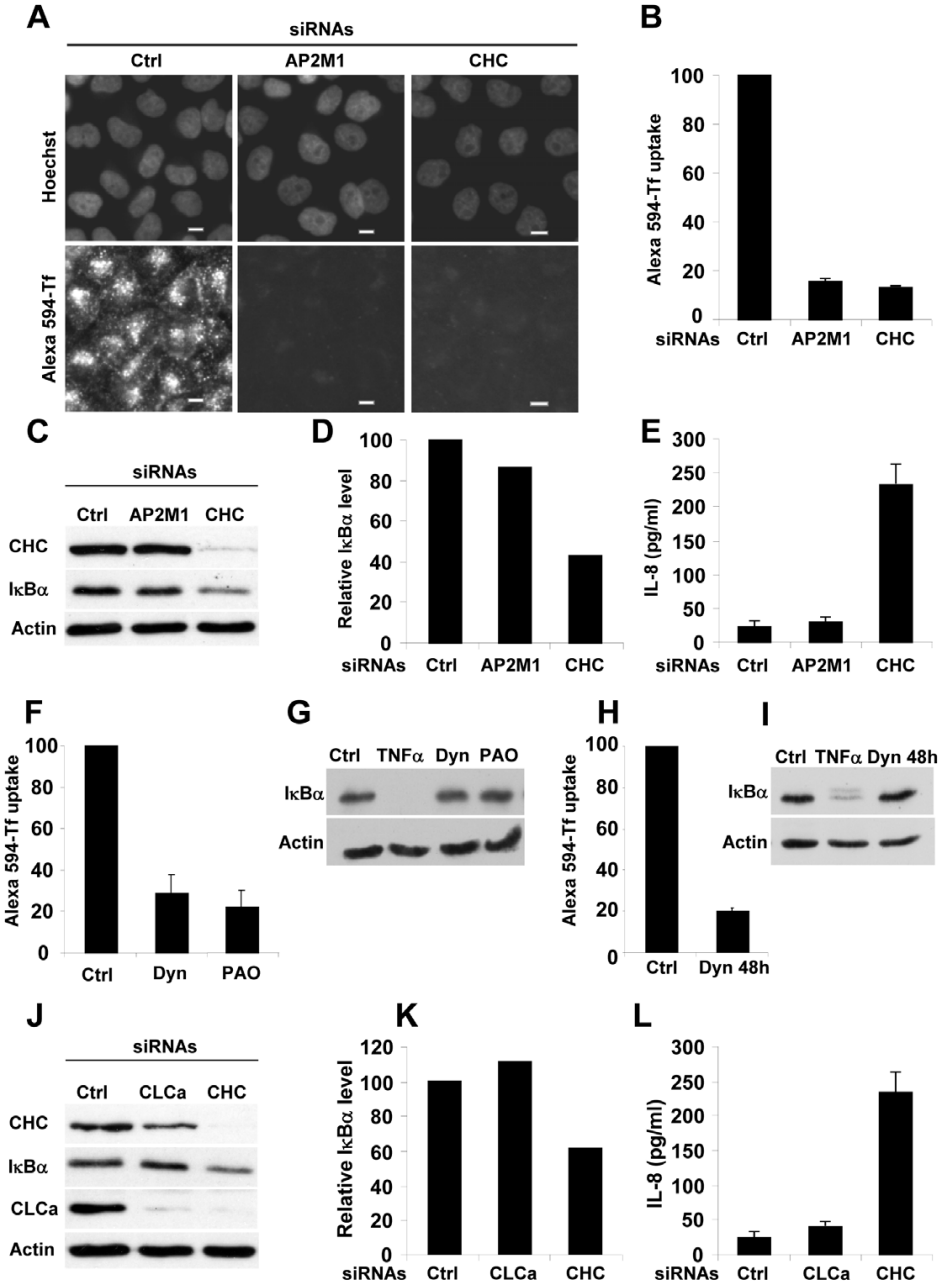
CHC regulates NF-κB activation independently of endocytosis and CLCa. (**A**) Uptake of Alexa 594-transferrin (Alexa 594-Tf) in cells transfected with control (left panels), AP2M1 (middle panels) or CHC (right panels) siRNAs; Scale bars, 10 µm. (**B**) Quantification of transferrin uptake by automated image analysis (results are expressed as the mean ± SD of 12 images; graph representative of 2 independent experiments). (**C**) AP2M1 knockdown fails to enhance IκBα degradation. Cell lysates from control, AP2M1 or CHC siRNA-transfected cells were analyzed by immunoblotting using indicated antibodies. Actin is shown as a loading control. (**D**) Densitometric quantification of the levels of IκBα shown in Figure 4C (graph representative of 2 independent experiments). (**E**) AP2M1 knockdown fails to induce constitutive IL-8 expression. HeLa cells were transfected with control, AP2M1 or CHC siRNAs for 72 hours. Supernatants were collected to measure the concentration of IL-8 by ELISA (results are expressed as the mean ± SD of 3 independent experiments). (**F**) Inhibition of transferrin uptake after dynasore and PAO treatment. HeLa cells were left untreated (Ctrl) or treated with dynasore (80 µM) (Dyn) or PAO (5 µM) 10 minutes before and during the transferrin uptake assay (results are expressed as the mean ± SD of 18 images; graph representative of 2 independent experiments). (**G**) Dynasore and PAO fail to enhance basal degradation of IκBα. HeLa cells were pretreated for 10 minutes with dynasore (80 µM) or PAO (5 µM) and analyzed by western immunoblotting using an IκBα antibody. Actin is shown as a loading control (results representative of 2 independent experiments). (**H**) Long-term inhibition of endocytosis in dynasore-treated HeLa cells. Transferrin uptake in HeLa cells left untreated or treated with dynasore (80 µM) for 48 hours (results are expressed as the mean ± SD of 18 images; graph representative of 2 independent experiments). (**I**) Long-term inhibition of endocytosis fails to enhance the basal degradation of IκBα. Basal degradation of IκBα in HeLa cells left untreated or treated with dynasore (80 µM) for 48 hours. As positive control of the degradation of IκBα, cells were stimulated for 20 minutes with TNFα (results representative of 2 independent experiments). (**J**) CLCa knockdown fails to enhance IκBα degradation. Cell lysates from control, CLCa or CHC siRNA transfected cells were analyzed by immunoblotting using indicated antibodies. Actin is shown as loading control. (**K**) Densitometric quantification of IκBα levels shown in Figure 4F (Graph representative of 2 independent experiments). (**L**) CLCa knockdown fails to induce constitutive IL-8 expression. HeLa cells were transfected with control, CLCa or CHC siRNAs for 72 hours and supernatants were collected to measure the concentration of IL-8 by ELISA (results are expressed as the mean ± SD of 3 independent experiments).

Through its activity in CME, CHC is involved in the internalization of nutrients, pathogens, antigens, growth factors and receptors [13], [15], [16]. To test whether CHC regulated indirectly the NF-κB pathway via its function in CME, we measured p65 nuclear translocation and IL-8 secretion in cells where CME was disrupted by RNAi-mediated depletion of the μ2-subunit of the main CME adaptor protein AP-2 (AP2M1). The recruitment of AP-2 at the plasma membrane is critical for the initiation of CME [23]. AP-2 interacts with sorting signals present in the cytoplasmic domains of membrane proteins destined to become cargo in the coated vesicles. In addition, AP-2 recruits clathrin onto the membrane, where it functions as a scaffold for vesicle budding. First, in order to demonstrate that CME was impaired in AP2M1 and CHC-depleted cells, the CME-dependent mechanism of transferrin uptake was monitored in HeLa cells. As previously reported [23], depletion of both CHC and AP2M1 impaired the uptake of fluorescently labeled transferrin (Figures 4A and 4B). However, although the depletion of AP2M1 blocked transferrin uptake to the same extent as CHC knockdown, it failed to increase basal IκBα degradation (Figures 4C and 4D) and IL-8 secretion (Figure 4E), suggesting that CHC controls basal NF-κB activation and gene expression independently of its activity in CME. In order to further validate this result, we tested whether chemical inhibition of endocytosis by the drugs phenylarsine oxide (PAO) and dynasore had an effect on NF-κB signaling. PAO is a chemical compound that, at low micromolar concentrations, blocks CME [24]. Dynasore is a cell-permeable small molecule that inhibits the GTPase activity of dynamin and blocks the formation of clathrin-coated vesicles [25]. Whereas a short term incubation with these drugs effectively blocked transferrin uptake (Figure 4F), neither of them had an effect on the degradation of IκBα (Figure 4G). HeLa cells stimulated with the inflammatory cytokine tumor necrosis factor α (TNFα) that rapidly activates NF-κB, were used as positive control for the degradation of IκBα. To better mimic the long-lasting effect of CHC knockdown on endocytosis, endocytosis and IκBα degradation were examined after 48 hours of dynasore treatment. Although endocytosis was still effectively blocked (Figure 4H), the degradation of IκBα was unchanged compared to untreated cells (Figure 4I), showing that inhibition of endocytosis had no effect on the activation of NF-κB. Taken together, these results strongly indicated that CHC regulates basal NF-κB activation independently of its function in endocytosis.

CHC is associated to CLCs in clathrin triskelion structures. The functional roles of CLCs have been recently characterized using RNAi. Knocking down CLCs has no effect on CME or the formation of clathrin-coated pits [26]. However, it causes alterations in protein trafficking at the TGN resulting from disruption of huntingtin interacting protein 1 related (HIPR1) recruitment to clathrin-coated structures and disorganization of the actin cytoskeleton [26]. Since CLCs are unstable unless they are bound to CHC [27], we tested whether the effect of CHC depletion on NF-κB activation was indirectly due to CLC degradation. For this purpose, IκBα degradation and IL-8 secretion were analyzed in HeLa cells depleted of the protein clathrin light chain a (CLCa) by RNAi. Although the degree of CLCa depletion detected by western immunoblotting was similar in cells transfected with CHC and CLCa siRNAs (Figure 4J), knocking down CLCa failed to induce constitutive IκBα degradation (Figures 4J and 4K) and IL-8 secretion (Figure 4L). These results showed that the effects of CHC depletion on NF-κB signaling were directly caused by the depletion of CHC and not by the associated depletion of CLCa. Altogether, these results strongly indicated that CHC prevents constitutive NF-κB activation independently of endocytosis and clathrin light chains.

## Discussion

Most research groups investigating the regulation of NF-κB activation have focused their studies on the mechanisms induced after cell exposure to various stimuli, including inflammatory cytokines and microbial products. In particular, the canonical pathway of NF-κB activation that depends on the phosphorylation and degradation of IκB proteins downstream of the activation of the IKK complex has been well characterized. In contrast, although constitutive NF-κB activation has been associated with inflammatory disorders and numerous cancers [3], [28], the mechanisms that lead to elevated basal NF-κB activation remain unclear.

Here we show that CHC functions as a built-in molecular brake that ensures a tight control of basal NF-κB activation and gene expression by preventing constitutive nuclear localization of p65 in absence of stimulation. Using RNAi to reduce cellular levels of CHC, we found that CHC is required for the proper spatial regulation of p65 in unstimulated epithelial cells. Whereas p65 was almost exclusively localized in the cytoplasm of control cells, both cytoplasmic and nuclear localization was observed in cells depleted of CHC. The localization of p65 is largely dependent on IκB proteins that sequestrate the transcription factor in the cytoplasm. Genetic deletion or mutations of IκBα lead to constitutive nuclear localization and NF-κB activation [11], [28]. In line with these studies, we found that the effect of CHC depletion on p65 was associated with a strong reduction in the level of IκBα suggesting that constitutive p65 nuclear localization was likely due to reduced IκBα level. Quantification of IκBα mRNA by quantitative real time PCR revealed that the level of *IκBα* mRNA was slightly elevated in CHC depleted cells. This result, which can be explained by the fact that *IκBα* is a target gene of NF-κB that is upregulated by constitutive NF-κB activation, indicated that the reduction of IκBα found in CHC depleted cells, was not due to transcriptional inhibition but to elevated basal IκBα degradation. This hypothesis was further supported by data showing that the knockdown of CHC induced constitutive phosphorylation of IκBα at position 32, a phosphorylation event critical to target IκBα for rapid degradation via the ubiquitin proteasome pathway. The role of CHC in the control of basal NF-κB activation was functionally relevant. Indeed a strong induction of IL-8, whose expression is controlled by NF-κB, was observed in cells depleted of CHC showing that, via its activity on NF-κB, CHC participates to gene regulation. Interestingly, a role of CHC in p53-mediated transcription has been recently reported. Enari et al. showed that a fraction of CHC proteins that localize to the nucleus bind to p53-responsive promoters and favor transcription by stabilizing p53 interactions with proteins such as the histone acetyltransferase p300 [19]. Although CHC contributes to p53 and NF-κB-regulated gene expression by different mechanisms, our data provide a second set of evidence for a role of CHC in gene regulation.

Via its role in CME, CHC is involved in many cellular processes including intracellular trafficking of receptors and nutrient uptake [13]. To investigate whether CHC indirectly affected basal NF-κB activation via its implication in CME, we analyzed the level of IκBα and IL-8 expression in conditions where CME was disrupted by the depletion of the μ2-subunit of the main CME adaptor AP-2 or by chemical inhibition. Whereas the CME-dependent process of transferrin uptake was almost completely abolished, AP2M1 depletion or treatment with the endocytosis inhibitors PAO and dynasore had no effect on the activation of NF-κB signaling, suggesting that CHC was involved in this pathway independently of endocytosis. As CLCs are unstable unless bound to CHC, the knockdown of CHC is associated with cellular depletion of CLCs. Interestingly, we showed that the depletion of CLCa has no effect on IκBα degradation and IL-8 expression, indicating that CHC alone contributes to the regulation of the NF-κB pathway. It also suggested that CHC functions in this pathway independently of the roles that CHC and CLCa share in protein trafficking at the TGN [26].

A raising number of proteins involved at different levels of the signaling pathway contribute to the tight control of basal NF-κB activation in resting cells [3]. For instance, expression of the tumor suppressor Gprc5a prevents constitutive p65 nuclear localization and NF-κB activation [29]. Although the mechanism remains unclear, the authors propose that the presence of Gprc5a may promote the stabilizing interaction between β-arrestin and IκBα. The role of silencer of death domain (SODD) has also been reported [30]. SODD deficiency leads to an increase of NF-κB activation and cytokine expression in absence of stimulation. This protein functions as a gatekeeper that constitutively associates with the cytoplasmic death domain of TNF receptors (TNFR) and blocks TNFR signaling in the absence of ligand. Constitutive activity of NF-κB was also described in c-Abl null fibroblasts [31]. In contrast to previous mechanisms, unstimulated fibroblasts did not exhibit an increase in IκBα degradation or p65 nuclear translocation but reduced levels of the negative regulator histone deacetylase HDAC1. The mechanism by which CHC functions in NF-κB signaling remains to be elucidated. Based on our data, we propose that this protein acts as a built-in molecular break that prevents the spontaneous activation of the IKK complex. This hypothesis is supported by the observation that constitutive IκBα degradation and IL-8 secretion were almost completely abolished when IKKα was depleted, and that basal IκBα phosphorylation was enhanced after CHC knockdown. Because the localization of IKKs is critical for their activity [32], the implication of CHC in the sub-cellular distribution of the IKK complex should be further investigated.

We also report here that CHC impedes constitutive IL-8 secretion by unstimulated HeLa and MCF-7 cells. It is well established that constitutive IL-8 secretion by epithelial cells can lead to chronic recruitment of macrophages that produce and secrete into the microenvironement a variety of cytokines, chemokines and growth factors involved in inflammation-related diseases such as inflammatory bowel disease. In addition, some of these factors promote angiogenesis and act directly on epithelial cells to favor adenoma formation and progression to adenocarcinomas [1]. Therefore by showing that CHC prevents constitutive expression of the proinflammatory and tumorigenic factor IL-8, our data suggest that alterations in CHC expression may be associated with chronic inflammation disorder or cancer. As a consequence, CHC expression, that can be regulated by external stimuli such as androgens [33], should be systematically investigated in tumors and inflamed tissues.
